# Clinical characteristics and survival outcomes of extrapulmonary neuroendocrine carcinomas: a retrospective study

**DOI:** 10.3389/fendo.2025.1635630

**Published:** 2025-10-27

**Authors:** Junhao Xu, Yuanyuan Li, Benjie Xu, Jie Lian, Haibo Lu

**Affiliations:** Department of Outpatient Chemotherapy, Harbin Medical University Cancer Hospital, Harbin, China

**Keywords:** extrapulmonary neuroendocrine carcinoma, survival analysis, CEA, NSE, prognostic factors

## Abstract

**Background:**

Extrapulmonary neuroendocrine carcinomas (EPNECs) are rare, heterogeneous, and aggressive malignancies with limited evidence to guide management. This study aimed to investigate the clinical characteristics, prognostic factors, and treatment outcomes of EPNEC patients.

**Methods:**

We retrospectively analyzed 343 EPNEC patients treated at Harbin Medical University Cancer Hospital from May 2011 to December 2023. Data on demographics, primary tumor sites, tumor markers (CEA and NSE), treatments, and survival were collected. Kaplan–Meier and Cox proportional hazards models were used to evaluate prognostic factors, and subgroup analyses were performed for treatment modalities.

**Results:**

The median overall survival (OS) for the cohort was 23.7 months. Prognosis varied significantly by primary site, with genitourinary tumors showing the most favorable outcomes and hepatopancreatobiliary tumors the poorest. Independent predictors of worse survival included advanced stage (HR = 1.94, *p* < 0.001), lymph node metastasis (HR = 1.48, *p* = 0.02), elevated CEA (HR = 1.49, *p* = 0.04), and elevated NSE (HR = 1.48, *p* = 0.03). Patients with both CEA and NSE levels elevated had the shortest OS (*p* < 0.0001). Treatment effects were stage-specific: surgery improved survival only in stage I/II patients (HR = 0.26, *p* = 0.01), whereas chemotherapy (HR = 0.67, *p* = 0.02) and radiotherapy (HR = 0.45, *p* < 0.001) provided significant benefits in stage III/IV patients. Radiotherapy showed consistent benefit across most subgroups, including those with elevated biomarkers.

**Conclusion:**

EPNEC prognosis is influenced by tumor site, stage, lymph node involvement, and biomarker levels. Surgery is optimal for early-stage disease (I/II), while chemotherapy and radiotherapy provide survival benefits in advanced-stage (III/IV) patients. Combined CEA and NSE elevation indicates a particularly poor prognosis. These findings highlight the importance of individualized, stage- and biomarker-driven therapeutic strategies for EPNECs.

## 1 Introduction

Neuroendocrine neoplasms (NENs) are a diverse group of cancers primarily originating from neuroendocrine cells in the gastrointestinal and bronchopulmonary systems (1). These tumors are characterized by neuroendocrine features, including the secretion of peptides through autocrine or paracrine mechanisms that may stimulate tumor growth. According to the World Health Organization (WHO) classification, NENs are subdivided into well-differentiated neuroendocrine tumors (NETs), graded as G1 (Ki-67 <3% or <2 mitoses/10 high-power fields[HPF]), G2 (Ki-67 3–20% or 2–20 mitoses/10 HPF), and G3 (Ki-67 >20% with well-differentiated morphology), and poorly differentiated neuroendocrine carcinomas (NECs), which are inherently high-grade (G3) with small- or large-cell morphology. Both NETs and NECs most commonly arise in the gastrointestinal tract, pancreas, and lungs (2). As a subset of NENs, NECs are characterized by aggressive biological behavior, poor differentiation, and an overall unfavorable prognosis. The majority of NECs arise in the pulmonary system, most commonly presenting as small-cell carcinomas (3). However, a smaller proportion originates outside the lungs, referred to as extrapulmonary NECs (EPNECs), which pose unique clinical challenges due to their rarity and heterogeneity. According to a recent comparative analysis from the National Cancer Institute’s Surveillance, Epidemiology, and End Results (SEER) database, 8.7% of NECs are classified as EPNECs (3).

EPNECs are rare and aggressive tumors with a poor prognosis, and their epidemiological characteristics have gained increasing attention in recent years. Population-based studies from the Netherlands and the United States have reported a rising incidence of EPNECs, most commonly originating in the bladder and gastrointestinal tract, with survival strongly dependent on disease stage (4, 5). Given their pathological similarities to small-cell lung cancer (SCLC), serum biomarkers such as CEA and NSE may also hold prognostic value in EPNECs, although evidence remains limited (6). International guidelines, including those from the European Neuroendocrine Tumor Society (ENETS) and the National Comprehensive Cancer Network (NCCN), provide recommendations for the diagnosis, staging, and treatment of NENs, emphasizing the importance of tumor differentiation, stage, and primary site in guiding therapeutic decisions (7, 8). Although significant progress has been made in the treatment of NENs, including targeted therapy and peptide receptor radionuclide therapy (PRRT), chemotherapy continues to play a central role in poorly differentiated NECs (9, 10). For EPNECs, therapeutic strategies remain highly dependent on the primary tumor site and often involve a combination of surgery, chemotherapy, radiotherapy, or targeted therapy (11).

Overall, the incidence of EPNECs is rising globally, with prognosis influenced by tumor site, stage, and biomarkers such as CEA and NSE. Given the rarity and heterogeneity of EPNECs, optimal treatment strategies remain unclear. This study aims to evaluate the clinical characteristics, prognostic factors, and impact of different treatment modalities on survival in EPNEC patients.

## 2 Materials and methods

We retrospectively collected the medical records of patients diagnosed with EPNEC at Harbin Medical University Cancer Hospital from May 2011 to December 2023. A graphical abstract summarizing the overall study design and analysis workflow is provided in the **Supplementary Materials** (**Supplementary Figure A**). All included patients were 18 years or older and had no concurrent malignancies of other types. For each patient, the following data were collected: age, gender, primary tumor site, primary tumor size, lymph node metastasis, tumor staging, levels of tumor markers (CEA and NSE) at the time of diagnosis, treatment modalities, and overall survival (OS). The size of the primary tumor and the presence of lymph node metastasis were determined based on imaging data or postoperative pathology. Tumor staging was performed according to the 8th edition of the AJCC Cancer Staging Manual. The normal reference values for tumor markers CEA and NSE were 0–5 ng/ml and 0–15.2 ng/ml, respectively, according to the laboratory standards at our institution. OS was defined as the time from the date of diagnosis to the date of death or the last follow-up. Other prognostic factors were also analyzed. Patients who received chemotherapy were all treated with platinum-based or taxane-based combination regimens. Demographic and tumor characteristics were expressed as frequencies (percentages) for categorical variables, and group differences were compared using the Pearson chi-square test. Survival was analyzed using the Kaplan-Meier method with log-rank tests. Univariate and multivariate Cox proportional hazards models were used to estimate hazard ratios (HRs) for OS. Adjusted hazard ratios (aHRs) controlling for age, sex, stage, lymphatic status, and serum biomarkers (CEA and NSE) were calculated, and subgroup analyses were performed based on these adjusted models, examining different treatment strategies. A two-sided *p* value <0.05 was considered statistically significant. All analyses and plots were conducted in R version 4.2.2.

This study was conducted according to the Declaration of Helsinki and approved by the Medical Ethics Committee of Harbin Medical University Cancer Hospital. Given its retrospective nature, the requirement for informed consent was waived.

## 3 Results

### 3.1 Clinical and demographic baseline characteristics

**Figure 1:**
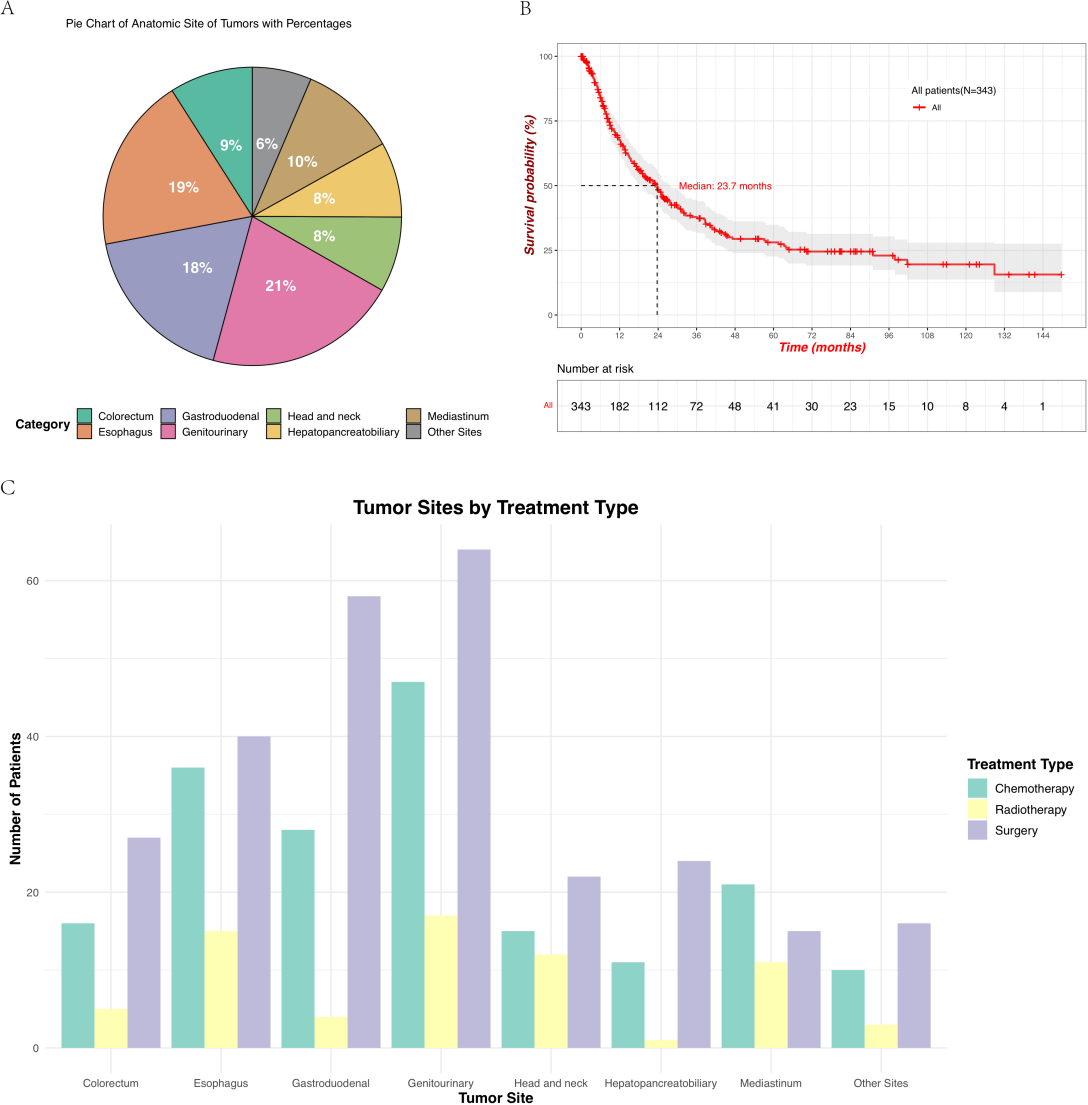
**(A)** Distribution of the primary tumor sites in patients with EPNECs, shown as a pie chart with percentages. Due to rounding, the total may not add up to 100%. **(B)** Kaplan–Meier survival curve showing OS for all 343 patients, with the number at risk displayed below the plot. **(C)** Treatment modalities across different tumor sites include chemotherapy, radiotherapy, and surgery.

A total of 343 patients with EPNECs across diverse anatomic sites were included in this retrospective cohort (**Table 1**). The distribution of cases was as follows: genitourinary (72, 21.0%), esophagus (65, 18.9%), gastroduodenal (61, 17.8%), mediastinum (36, 10.5%), colorectal (31, 9.0%), head and neck (28, 8.2%), hepatopancreatobiliary (28, 8.2%), and other sites (22, 6.4%) (**Figure 1A**). The “Other Sites” category includes the abdominal cavity, pelvic cavity, chest wall, brain, and other locations. Among the 343 diagnosed cases of EPNEC, the median age was 59 years, with 221 male patients (64.5%). Significant heterogeneity in demographic, clinicopathological, and therapeutic features was observed across anatomic sites.

**Table 1:** Patient characteristics and treatment patterns.

	Anatomic site of tumor (N=343)								
	Genitourinary	Esophagus	Gastroduodenal	Mediastinum	Colorectum	Head and neck	Hepatopancreatobiliary	Other Sites	*p*
**All patients [n (%)]**	72 (100)	65 (100)	61 (100)	36 (100)	31 (100)	28 (100)	28 (100)	22 (100)	
**Age**									***0.016***
<59	48 (66.7)	30 (46.2)	23 (37.3)	13 (36.1)	14 (45.2)	14 (50.0)	14 (50.0)	7 (31.8)	
≥59	24 (33.3)	35 (53.8)	38 (62.3)	23 (63.9)	17 (54.8)	14 (50.0)	14 (50.0)	15 (68.2)	
**Sex**									***< 0.001***
Male	12 (16.7)	60 (92.3)	52 (85.2)	21 (58.3)	22 (71.0)	20 (71.4)	16 (57.1)	18 (81.8)	
Female	60 (83.3)	5 (7.7)	9 (18.4)	15 (41.7)	9 (29.0)	8 (28.6)	12 (42.9)	4 (18.2)	
**Tumor Size**									***<0.001***
<3cm	31 (43.1)	13 (20.0)	10 (16.4)	13 (36.1)	16 (51.6)	21 (75.0)	13 (46.4)	17 (77.3)	
≥3cm	41 (56.9)	52 (80.0)	51 (83.6)	23 (63.9)	15 (48.4)	7 (25.0)	15 (53.6)	5 (22.7)	
**Lymphatic metastasis**									***< 0.001***
Yes	13 (18.1)	42 (64.6)	41 (67.2)	15 (41.7)	9 (29.0)	6 (21.4)	7 (25.0)	7 (31.8)	
No	59 (81.9)	23 (35.4)	20 (32.8)	21 (58.3)	22 (71.0)	22 (78.6)	21 (75.0)	15 (68.2)	
**Stage**									***<0.001***
I	32 (44.4)	7 (10.8)	0 (0)	2 (5.6)	2 (6.5)	5 (17.9)	1 (3.6)	2 (9.1)	
II	10 (13.9)	6 (9.2)	11 (18.0)	0 (0)	5 (16.1)	5 (17.9)	9 (32.1)	2 (9.1)	
III	19 (26.4)	23 (35.4)	32 (52.5)	4 (11.1)	11 (35.5)	5 (17.9)	4 (14.3)	2 (9.1)	
IV	11 (15.3)	29 (44.6)	18 (29.5)	30 (83.3)	13 (41.9)	13 (46.3)	14 (50.0)	16 (72.7)	
**Surgery**									***<0.001***
Yes	64 (88.9)	40 (61.5)	58 (95.1)	15 (41.7)	27 (87.1)	22 (78.6)	24 (85.7)	16 (72.7)	
No	8 (11.1)	25 (38.5)	3 (4.9)	21 (58.3)	4 (12.9)	6 (21.4)	4 (14.3)	6 (27.3)	
**Radiotherapy**									***<0.001***
Yes	17 (23.6)	15 (23.1)	4 (6.6)	11 (30.6)	5 (16.1)	12 (42.9)	1 (3.6)	3(13.6)	
No	55 (76.4)	50 (76.9)	57 (93.4)	2 5 (69.4)	2 6 (83.9)	16 (57.1)	27 (96.4)	19 (86.4)	
**Chemotherapy**									***< 0.001***
Yes	47 (65.3)	36 (55.4)	28 (45.9)	21 (58.3)	16 (51.6)	15 (53.6)	11 (39.3)	10 (45.5)	
No	25 (34.7)	29 (44.6)	33 (54.1)	15 (41.7)	15 (48.4)	13 (46.4)	17 (60.7)	12 (54.5)	
**CEA**									0.125
Normal	67 (93.1)	53 (81.5)	53 (86.9)	30 (83.3)	29 (93.5)	28 (100)	23 (82.1)	18 (81.8)	
Elevated	5 (6.9)	12 (18.5)	8 (13.1)	6 (16.7)	2 (6.5)	0 (0)	5 (17.9)	4 (18.2)	
**NSE**									***<0.001***
Normal	62 (86.1)	34 (52.3)	56 (91.8)	12 (33.3)	25 (80.6)	25 (89.3)	20 (71.4)	16 (72.7)	
Elevated	10 (13.9)	31 (47.7)	5 (8.2)	24 (66.7)	6 (19.4)	3 (10.7)	8 (28.6)	6 (27.3)	

Data are represented as *n* (%).

*p* values werecalculated using chi-square or Fisher's exact test for categorical variables; <0.05 was considered statistically significant.

CEA, carcinoembryonic antigen; NSE, neuron-specific enolase.

Bold values were considered statistically significant.

Patients aged ≥ 59 years were predominant in gastroduodenal (62.3%), mediastinal (63.9%), and colorectal tumors (54.8%), while more than half of the cases in the genitourinary had an onset age younger than the median age (66.7%) (*p* = 0.016). Sex distribution varied markedly (*p* < 0.001), with male predominance in esophageal (92.3%), gastroduodenal (85.2%), and head/neck tumors (71.4%), whereas females only constituted higher proportions in genitourinary (83.3%). Tumors ≥3 cm were more frequent in esophageal (80.0%) and gastroduodenal sites (83.6%) (*p* < 0.001). Lymph node metastasis was more commonly observed in the esophagus (64.6%) and gastroduodenal tumors (67.2%) (*p* < 0.001). Advanced-stage (IV) disease predominated in mediastinal (83.3%), whereas stage I tumors were most common in the genitourinary cohort (44.4%) (*p* < 0.001).

**Figure 2:**
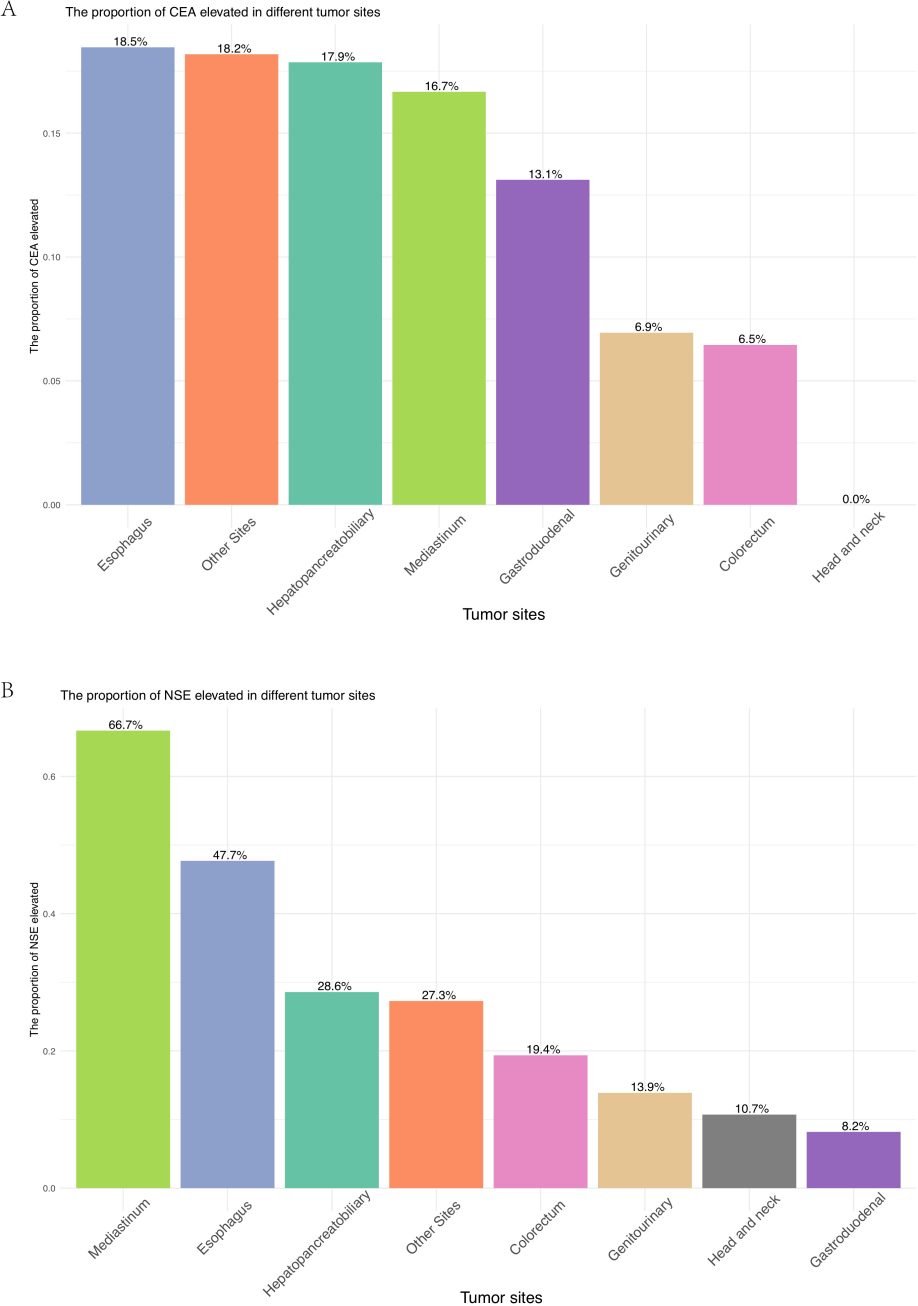
**(A)** Proportion of patients with elevated CEA across different primary tumor sites. **(B)** Proportion of patients with elevated NSE across different primary tumor sites.

For patients at all tumor sites, the number of patients who underwent surgery and chemotherapy was higher than those who received radiotherapy (**Figure 1C**, **Supplementary Figure B**). Surgical resection rates were highest in gastroduodenal (95.1%), genitourinary (88.9%), and colorectal tumors (87.1%) but lower in mediastinal (41.7%) and esophageal tumors (61.5%) (*p* < 0.001). Radiotherapy utilization peaked in head/neck (42.9%) and mediastinal tumors (30.6%), while being least frequent in hepatopancreatobiliary tumors (3.6%) (*p* < 0.001). Chemotherapy was most administered in genitourinary (65.3%) and mediastinal tumors (58.3%) (*p* < 0.001). Elevations in tumor markers also showed site-specific differences across various anatomical locations. Though no significant inter-site differences were observed in CEA elevation (*p* = 0.125), an increase in CEA was more commonly observed in esophagus tumors (18.5%), whereas no elevation in CEA was detected in head/neck tumors (**Figure 2A**). Elevated NSE levels were elevated in tumors from all sites, with the most significant prevalent in mediastinal tumors (66.7%) and esophageal (47.7%) (**Figure 2B**) (*p* < 0.001).

### 3.2 Survival analysis of different prognostic factors

**Figure 3:**
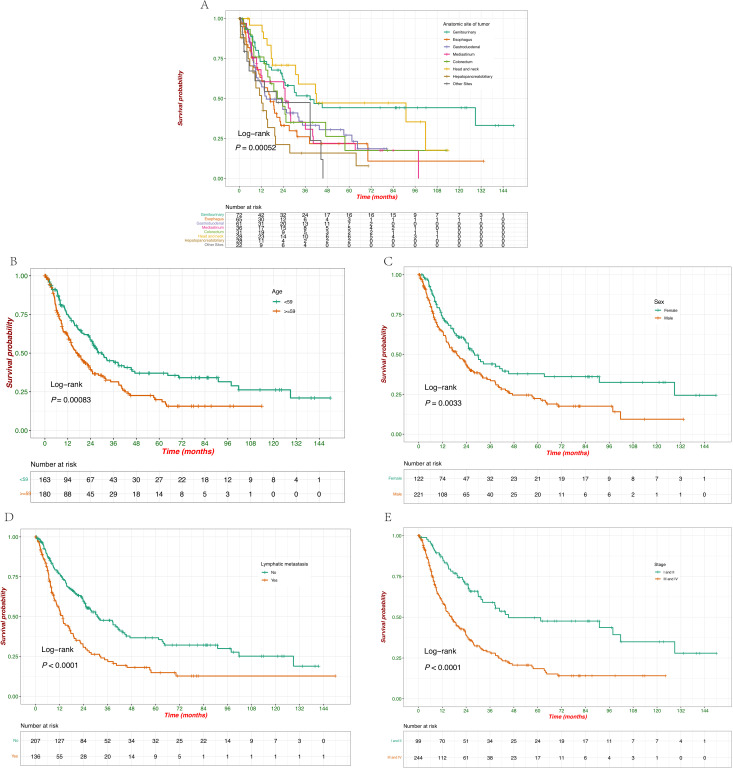
Kaplan–Meier survival curves of OS according to baseline clinicopathological characteristics. **(A)** Survival probabilities compared with the anatomic site of tumors. **(B)** Survival probabilities stratified by age (<59 vs. ≥59 years). **(C)** Survival probabilities stratified by sex (female vs. male). **(D)** Survival probabilities stratified by lymphatic metastasis at diagnosis (yes vs. no). **(E)** Survival probabilities stratified by clinical stage (I/II vs. III/IV).

The median OS (mOS) for all patients was 23.7 months (95% CI: 18.8 - 27.6 months; **Figure 1B**). Significant differences were observed across the groups based on the anatomical site of the tumor, age, sex, lymphatic metastasis, and disease stage. Survival probabilities varied significantly across different tumor sites, with patients having tumors in the genitourinary demonstrating better survival outcomes while tumors originating from the hepatopancreatobiliary had a poorer OS. The Log-rank test confirmed a significant difference in survival between these groups (*p* = 0.00052) (**Figure 3A**). Further analysis show that the 1-year, 3-year, and 5-year survival rates for each site were as follows: genitourinary (73.1%, 51.7%, 44.3%), esophagus (63.2%, 26.1%, 21.8%), gastroduodenal (59.1%, 33.3%, 27.1%), mediastinum (64.3%, 35.2%, 22.0%), colorectum (76.0%, 35.1%, 17.5%), head and neck (95.8%, 59.0%, 47.2%), hepatopancreatobiliary (51.9%, 15.9%, 15.9%), and other sites (61.1%, 47.5%, not reached). Younger patients (< 59 years) exhibited better survival compared to older patients (≥ 59 years), with a statistically significant difference observed (*p* = 0.00083) (**Figure 3B**). Additionally, female patients had a significantly higher survival probability than male patients (*p* = 0.0033), as demonstrated by the Kaplan-Meier curves (**Figure 3C**). Patients without lymphatic metastasis at the time of diagnosis showed better survival outcomes compared to those with metastasis, with a highly significant difference (*p* < 0.0001) (**Figure 3D**). Stage I and II patients had a significantly better survival rate compared to stage III and IV patients (*p* < 0.0001) (**Figure 3E**), highlighting the prognostic importance of disease stage. These findings underscore the importance of tumor site, age, sex, lymphatic metastasis, and disease stage as significant predictors of survival outcomes in patients with EPNECs.

**Figure 4:**
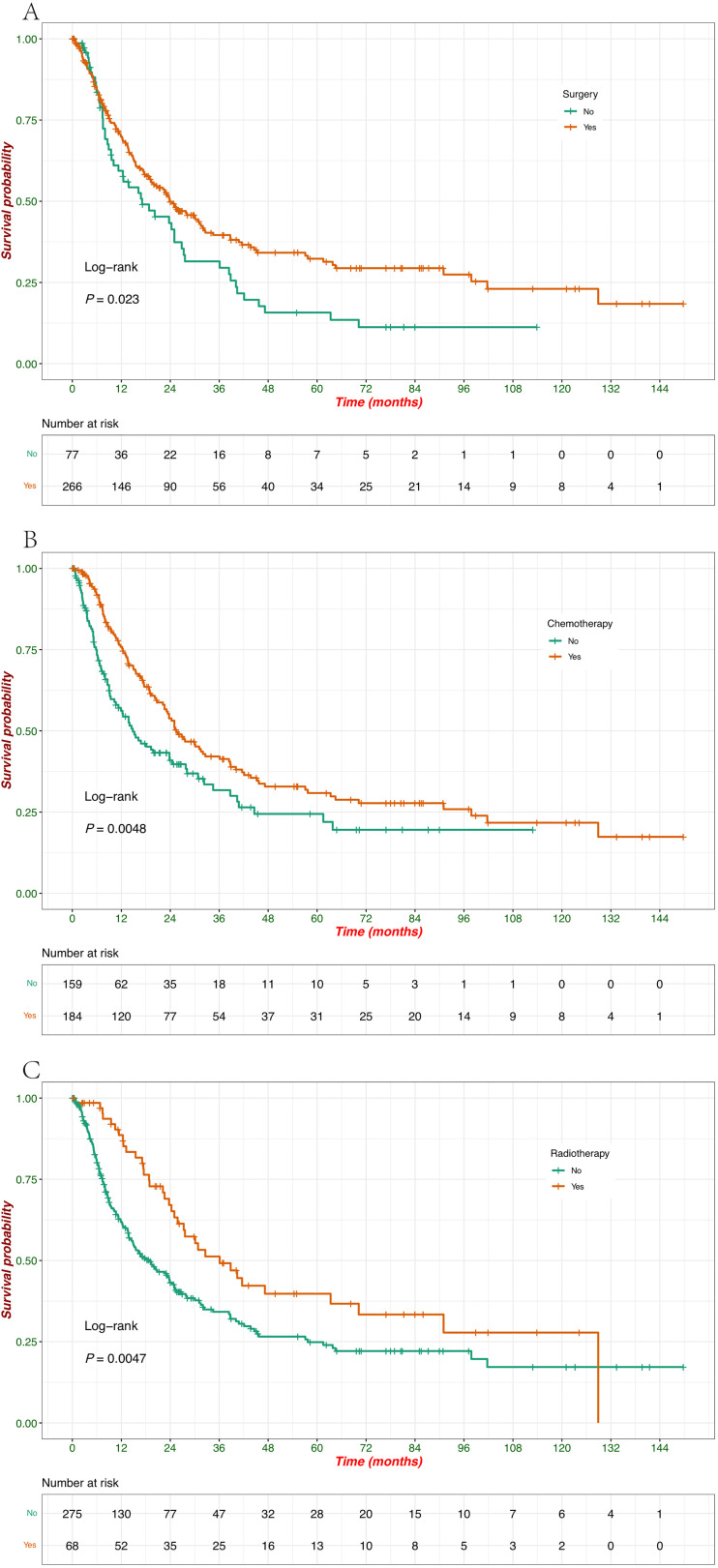
Kaplan–Meier survival curves of OS according to treatment modalities. **(A)** Survival probabilities stratified by patients treated with surgery (yes vs. no). **(B)** Survival probabilities stratified by patients treated with chemotherapy (yes vs. no). **(C)** Survival probabilities stratified by patients treated with radiotherapy (yes vs. no).

Compared to untreated patients, there was a significant impact of surgery, chemotherapy, and radiotherapy on survival, with each treatment modality showing a clear survival benefit (**Figures 4A–C**). Patients who underwent surgery had a significantly better survival probability than those who did not (*p* = 0.023) (**Figure 4A**). Surgical intervention positively affected patient outcomes, with the group undergoing surgery showing a prolonged survival period. Chemotherapy treatment was associated with improved survival, as evidenced by the significantly higher survival probability in patients who received chemotherapy than those who did not (*p* = 0.0048) (**Figure 4B**). These results suggest the beneficial role of chemotherapy in prolonging survival in this cohort. Similar to surgery and chemotherapy, radiotherapy also significantly improved survival outcomes (*p* = 0.0047) (**Figure 4C**). Patients who received radiotherapy exhibited a higher survival probability, indicating the positive effect of this treatment modality in extending patient survival.

**Figure 5:**
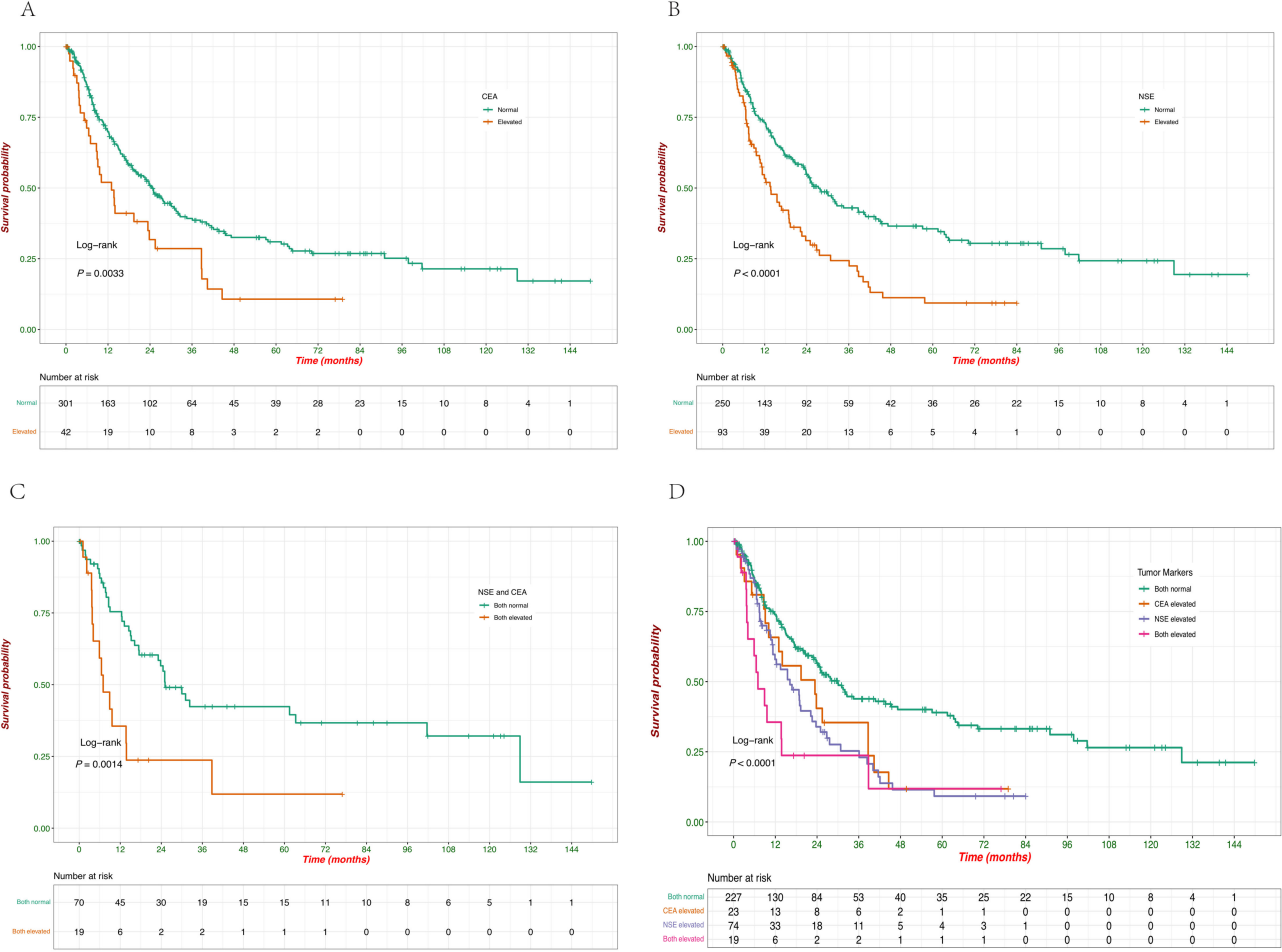
Kaplan–Meier survival curves of OS according to the levels of serum tumor markers. **(A)** Survival probabilities comparing patients with normal versus elevated CEA levels. **(B)** Survival probabilities comparing patients with normal versus elevated NSE levels. **(C)** Survival probabilities comparing patients with normal versus elevated combined NSE and CEA levels. **(D)** Survival probabilities among patients with different combinations of CEA and NSE levels (both normal, CEA elevated, NSE elevated, both elevated).

The impact of tumor marker levels (CEA, NSE) and their combinations on patient survival was analyzed using Kaplan-Meier survival curves. Patients with normal CEA levels had significantly better survival compared to those with elevated CEA levels (*p* = 0.0033), and the survival curves clearly show that elevated CEA is associated with a poorer prognosis (**Figure 5A**). Similarly, elevated NSE levels were associated with significantly reduced survival compared to normal NSE levels (*p* < 0.0001), indicating that elevated NSE is a strong negative prognostic factor in this cohort (**Figure 5B**). When considering both NSE and CEA levels together, patients with both markers elevated had the worst survival outcomes (*p* = 0.0014), which highlights the compounded effect of elevated tumor markers on survival (**Figure 5C**). The combined analysis of both tumor markers (CEA and NSE) demonstrated that patients with both markers normal had the best survival, followed by those with only one marker elevated. Patients with both CEA and NSE elevations had the poorest survival outcomes, with the difference being highly significant (*p* < 0.0001) (**Figure 5D**). When patients were grouped based on tumor size, and survival analysis was performed, no statistically significant differences in survival were observed (**Supplementary Figure C**).

### 3.3 Cox analyses of factors associated with survival

To further determine the impact of various factors on OS, Cox regression analysis was performed to evaluate the impact of factors on survival, both in univariate and multivariate models (**Table 2**). In the univariate analysis, age ≥59 years was associated with a significantly worse survival (HR = 1.63, 95% CI: 1.22-2.19, *p* < 0.001). However, in the multivariate model, age was not identified as an independent predictor of survival (aHR = 1.22, 95% CI: 0.81-1.54, *p* = 0.505). The male sex was associated with poorer survival in the univariate analysis (HR = 1.58, 95% CI: 1.16-2.15, *p* = 0.0036). However, in the multivariate analysis, this association was no longer significant (aHR = 1.15, 95% CI: 0.85-1.55, *p* = 0.378). The advanced stage (III/IV) was a strong negative prognostic factor in both univariate (HR = 2.50, 95% CI: 1.76–3.56, *p* < 0.001) and multivariate analyses (aHR = 1.94, 95% CI: 1.31–2.85, *p* < 0.001). Tumor size ≥3 cm did not show a significant association with survival in univariate analysis (HR = 0.93, 95% CI: 0.70-1.24, *p* = 0.6353). In the univariate analysis, surgery was associated with improved survival (HR = 0.69, 95% CI: 0.50-0.95, *p* = 0.0235), but this was not significant in the multivariate analysis (aHR = 0.77, 95% CI: 0.50-1.20, *p* = 0.249). Radiotherapy significantly improved survival in both univariate (HR = 0.60, 95% CI: 0.41-0.86, *p* = 0.0052) and multivariate analyses (aHR = 0.53, 95% CI: 0.35-0.80, *p* = 0.003). Chemotherapy was significantly associated with improved survival in both univariate (HR = 0.66, 95% CI: 0.49-0.88, *p* = 0.005) and multivariate analyses (aHR = 0.65, 95% CI: 0.46-0.91, *p* = 0.01). Lymphatic metastasis was a strong negative prognostic factor in both univariate (HR = 1.97, 95% CI: 1.48-2.63, *p* < 0.001) and multivariate analyses (aHR = 1.48, 95% CI: 1.07-2.04, *p* = 0.02). Elevated CEA levels were associated with worse survival in the univariate (HR = 1.77, 95% CI: 1.20-2.60, *p* = 0.0038) and multivariate analyses (aHR = 1.49, 95% CI: 1.02-2.22, *p* = 0.04). Elevated NSE levels also showed a significant negative impact on survival in both univariate (HR = 1.91, 95% CI: 1.41-2.59, *p* < 0.001) and multivariate analyses (aHR = 1.48, 95% CI: 1.07-2.04, *p* = 0.03). These findings underscore the clinical relevance of advanced stage, radiation, chemotherapy, lymphatic metastasis, and tumor marker levels (CEA and NSE) as independent factors influencing survival in patients with EPNECs.

**Table 2:** Univariate and multivariate cox regression analysis.

	Univariate Cox regression analysis			Multivariate Cox regression analysis		
	HR	95% CI	*p* value	aHR	95% CI	*p* value
Age						
<59	1.00					
≥59	1.63	1.22-2.19	< 0.001*	1.22	0.81-1.54	0.505
Sex						
Male	1.58	1.16-2.15	0.0036*	1.15	0.85-1.55	0.378
Female	1.00					
Stage						
I/II	1.00					
III/IV	2.50	1.76-3.56	<0.001*	1.94	1.31-2.85	<0.001*
Tumor Size						
<3cm	1.00					
≥3cm	0.93	0.70-1.24	0.6353			
Surgery						
Yes	0.69	0.50-0.95	0.0235*	0.77	0.50-1.20	0.249
No	1.00					
Radiotherapy						
Yes	0.60	0.41-0.86	0.0052*	0.53	0.35-0.80	0.003*
No	1.00					
Chemotherapy						
Yes	0.66	0.49-0.88	0.0050*	0.65	0.46-0.91	0.01*
No	1.00					
Lymphatic metastasis						
Yes	1.97	1.48-2.63	< 0.001*	1.48	1.07-2.04	0.02*
No	1.00					
CEA						
Normal	1.00					
Elevated	1.77	1.20-2.60	0.0038*	1.49	1.02-2.22	0.04*
NSE						
Normal	1.00					
Elevated	1.91	1.41-2.59	< 0.001*	1.48	1.07-2.04	0.03*

*p value < 0.05 was considered statistically significant. aHR, adjusted hazard ratio.

Bold values were considered statistically significant.

### 3.4 Subgroup analyses of treatment modalities

To further explore the robustness of treatment effects, we conducted subgroup analyses based on aHR derived from multivariable Cox proportional hazards models, with adjustment for other treatments, age, sex, stage, lymphatic metastasis, and serum biomarkers (CEA and NSE) as appropriate.

**Figure 6:**
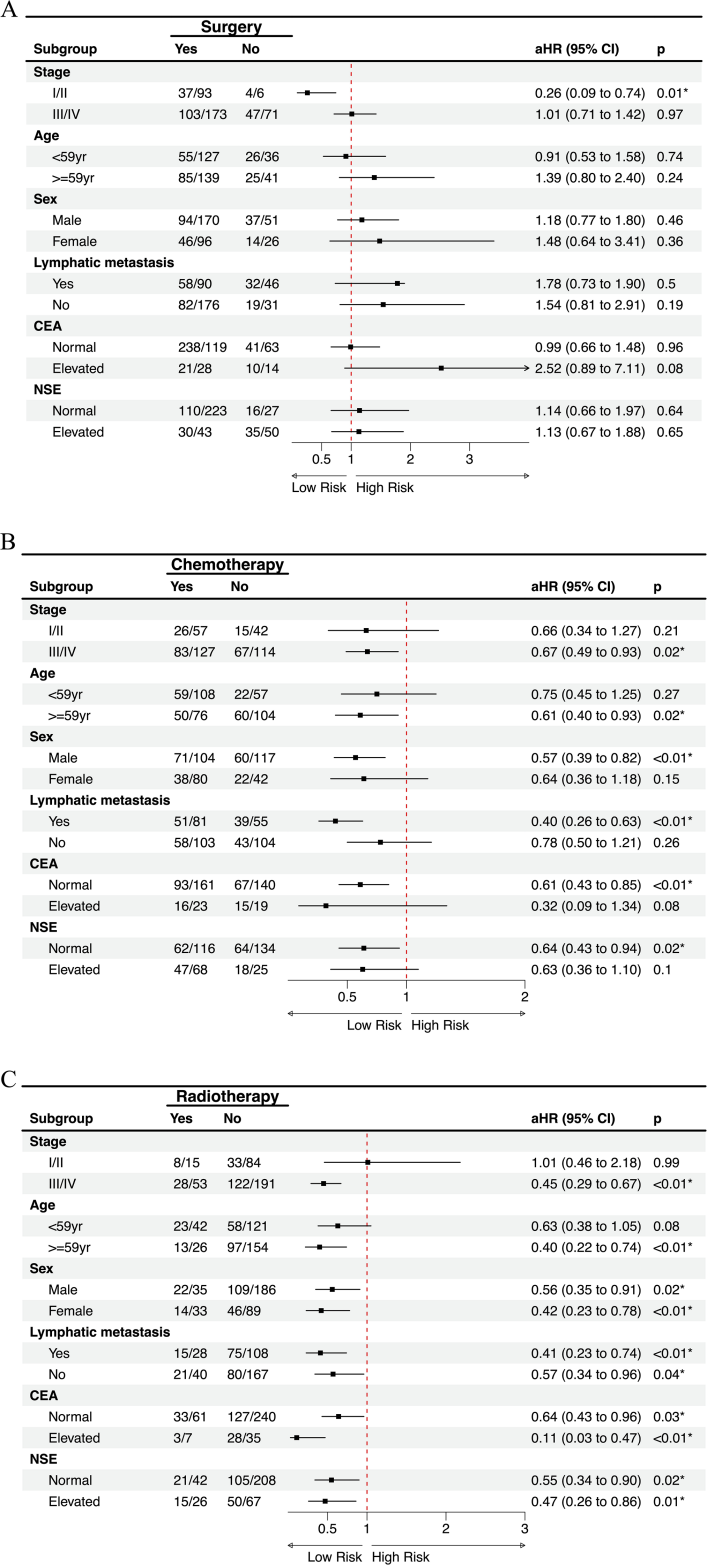
Subgroup analysis of treatment modalities on overall survival with multivariate adjustment. Forest plots showing the effects of **(A)** surgery, **(B)** chemotherapy, and **(C)** radiotherapy on overall survival across clinically relevant subgroups. Numbers indicate events/total patients in each subgroup. aHR, adjusted hazard ratio.

Surgery was associated with significantly improved OS in patients with stage I/II disease (aHR = 0.26, 95% CI 0.09–0.74, *p* = 0.01). In contrast, no survival advantage was observed in stage III/IV or other subgroups (**Figure 6A**). Chemotherapy provided significant benefit in stage III/IV patients (aHR = 0.67, 95% CI 0.49–0.93, *p* = 0.02), those aged ≥59 years (aHR = 0.61, 95% CI 0.40–0.93, *p* = 0.02), males (aHR = 0.57, 95% CI 0.39–0.82, *p* < 0.01), patients with lymphatic metastasis (aHR = 0.40, 95% CI 0.26–0.63, *p* < 0.01), and patients with normal CEA or NSE levels (both *p* < 0.05) (**Figure 6B**). Radiotherapy showed the broadest and most consistent survival benefits across subgroups, including stage III/IV patients (aHR = 0.45, 95% CI 0.29–0.67, *p* < 0.01), those aged ≥59 years (aHR = 0.40, 95% CI 0.22–0.74, *p* < 0.01), both sexes, patients with or without lymphatic metastasis, and those with normal or elevated tumor markers (all *p* < 0.05), with the strongest effect observed in patients with elevated CEA (aHR = 0.11, 95% CI 0.03–0.47, *p* < 0.01) (**Figure 6C**).

In summary, subgroup analyses based on multivariable Cox models demonstrated that surgery provided a marked survival advantage only in patients with stage I/II disease, but not in advanced stages or other subgroups. Chemotherapy was effective primarily in stage III/IV patients, older individuals, males, those with lymphatic metastasis, and patients with normal tumor markers, highlighting its selective benefit in high-risk groups. By contrast, radiotherapy yielded the most consistent and broad survival improvements across clinical and biomarker-defined subgroups, with particularly strong effects in patients with elevated CEA levels.

## 4 Discussion

The 2022 WHO classification of NENs emphasizes the distinction between well-differentiated NETs and poorly differentiated NECs, which is critical for accurate diagnosis, prognostic stratification, and guiding treatment decisions (2). NECs, particularly EPNECs, represent a rare and biologically aggressive subset of NENs, characterized by poor differentiation, high proliferative indices, and generally unfavorable prognosis. Given the rising global incidence of EPNECs and their aggressive behavior, there is an urgent need for comprehensive studies to improve diagnostic strategies, prognostication, and treatment approaches. Our study provides a comprehensive analysis of 343 EPNEC patients, integrating demographic, clinicopathological, biomarker, and treatment-related variables to delineate prognostic factors and survival outcomes.

Consistent with previous studies, EPNECs were observed across multiple anatomical sites, with the genitourinary system, esophagus, and gastroduodenal regions being the most frequent (12). Interestingly, our study revealed that genitourinary EPNECs presented at a younger age than tumors from other sites, aligning with prior population-based analyses (13–15). Survival outcomes varied significantly by tumor site, with genitourinary tumors demonstrating the most favorable prognosis and hepatopancreatobiliary tumors the worst. These findings corroborate prior reports highlighting the prognostic relevance of primary tumor location in EPNECs (3, 15–17). Age and sex were significant predictors of survival in univariate analyses; however, neither factor retained statistical significance in multivariate models, suggesting that their prognostic effects may be confounded by tumor stage, lymphatic metastasis, biomarker status, or treatment modality. Lymph node involvement has been consistently associated with poorer prognosis across various cancers, including EPNECs (18–20). In our study, lymphatic metastasis emerged as a strong independent negative prognostic factor, in line with previous findings in both EPNECs and other high-grade neuroendocrine malignancies, including SCLC (21–23). Moreover, lymph node metastasis was significantly more frequent in tumors originating in the esophagus and gastroduodenal regions, which may reflect the more aggressive behavior of these tumor types at diagnosis. The advanced stage (III/IV) was a strong independent predictor of poor survival, reinforcing the critical importance of early detection and accurate staging.

CEA and NSE, two well-established biomarkers in SCLC (24, 25), were evaluated for their diagnostic and prognostic value in EPNECs. In our study, we observed distinct differences in tumor marker levels among EPNECs originating from various anatomical sites. CEA elevation was more frequently seen in EPNECs from the esophagus, hepatopancreatobiliary, mediastinum, gastroduodenal, and other sites. In contrast, EPNECs originating from the genitourinary, colorectal, and head/neck regions showed relatively low rates of CEA elevation, with CEA elevation being absent in head/neck tumors. On the other hand, elevated NSE was common across most anatomical sites, particularly mediastinal EPNECs. Overall, NSE is frequently elevated in EPNECs, reflecting its utility in diagnosis and disease monitoring. Nonetheless, CEA retains an important role, particularly when NSE elevation is modest or absent. Evaluating both markers concurrently offers a more comprehensive assessment of EPNEC. Our survival analysis revealed that elevated CEA and NSE levels were associated with poorer prognosis, with the worst outcomes observed in patients exhibiting elevation of both markers. This combined elevation may reflect a more aggressive disease phenotype, highlighting the value of a multifaceted biomarker approach for prognostication in EPNECs.

Therapeutic strategies for EPNECs are largely extrapolated from SCLC (26), with international guidelines from ENETS and NCCN recommending multimodal treatment based on tumor differentiation, stage, and primary site (7, 8). In line with these recommendations, our cohort demonstrated differential survival benefits across treatment modalities, which were further clarified through subgroup analyses adjusted for age, sex, stage, lymphatic metastasis, and tumor markers. Surgery conferred a significant survival advantage primarily in patients with stage I/II disease, but not in stage III/IV patients, underscoring its role as a potentially curative option in early-stage EPNECs, which was consistent with data from other studies that highlight the benefit of surgery in early-stage disease (16, 27, 28). Chemotherapy provided a significant survival benefit in stage III/IV patients, older individuals, males, patients with lymphatic metastasis, and those with normal CEA or NSE levels, supporting its selective efficacy in high-risk or advanced-stage patients (29). Regarding chemotherapy regimens, prior studies have demonstrated that platinum-based and taxane-based combinations remain standard first-line options for poorly differentiated NENs, offering meaningful response rates and progression-free survival (9, 10, 29). Our cohort exclusively received these regimens, and the observed survival benefits in advanced-stage patients align with published evidence, emphasizing the continued relevance of systemic chemotherapy despite advances in targeted therapies and PRRT (9, 10). Radiotherapy exhibited the broadest and most consistent survival advantage across multiple clinical and biomarker-defined subgroups, including those with elevated CEA and NSE levels, suggesting its utility as an integral component of multimodal therapy, particularly for unresectable or advanced tumors.

This study has several limitations. The retrospective design from a single center introduces potential selection bias and confounding. Certain subgroups had limited sample sizes, which may reduce statistical power. Prospective, multicenter studies are needed to validate these findings and refine patient-specific therapeutic strategies. Additionally, the molecular profiling of EPNECs remains an area of active investigation, and future studies should explore the genetic and molecular characteristics of these tumors to provide more targeted therapeutic approaches.

## 5 Conclusion

In conclusion, EPNECs are clinically heterogeneous malignancies with survival influenced by tumor site, stage, lymphatic metastasis, tumor marker status, and treatment modality. Surgery offers substantial survival benefits in early-stage disease, whereas chemotherapy and radiotherapy are particularly beneficial in advanced-stage or high-risk patients. Elevated CEA and NSE are strong negative prognostic factors, and their combined assessment can guide risk stratification and treatment planning. Our findings provide a comprehensive framework for evidence-based management of EPNECs and underscore the need for individualized, stage- and biomarker-driven therapeutic strategies for this rare and aggressive cancer.

## Data Availability

The raw data supporting the conclusions of this article will be made available by the authors, without undue reservation.
