# Supplementary material for: Clinical characteristics and survival outcomes of extrapulmonary neuroendocrine carcinomas: a retrospective study
Source: Front Endocrinol (Lausanne). 2025 Oct 27;16:1635630. doi: 10.3389/fendo.2025.1635630 (PMC12597801; doi:10.3389/fendo.2025.1635630)
Supplement: Supplementary file 1 [file DataSheet1.docx]

**Supplementary Figure A:** The overall study design and analysis workflow

**
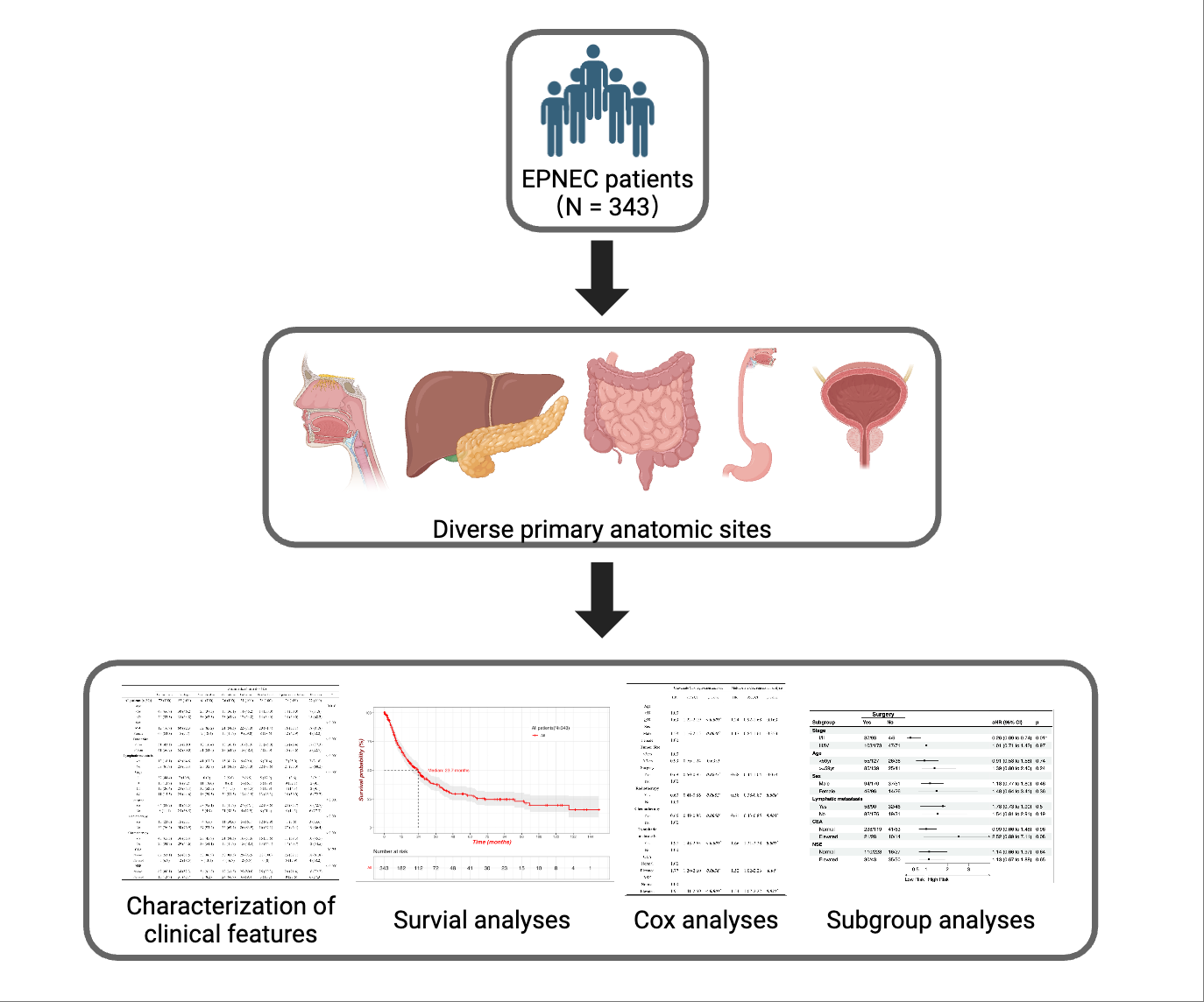
**

**Supplementary Figure B:** Stacked plot of treatment modalities across different tumor sites

**Supplementary Figure C:** Kaplan-Meier survival curves based on tumor size.
